# Recurrent adult-onset hypophyseal Langerhans cell histiocytosis after radiotherapy: a case report

**DOI:** 10.1186/1752-1947-6-341

**Published:** 2012-10-08

**Authors:** Ryan K Funk, Daniel J Ferraro, Richard J Perrin, Kyle M Hurth, John J Stephens, David B Mansur, Michael R Chicoine, Joseph R Simpson, Jerry J Jaboin

**Affiliations:** 1 Department of Radiation Oncology, Mallinckrodt Institute of Radiology and Siteman Cancer Center, Washington University Medical School, 4511 Forest Park Avenue, Saint Louis, MO, 63108, USA; 2 Division of Neuropathology, Department of Pathology and Immunology, Washington University School of Medicine, 660 South Euclid Avenue, Campus Box 8118, Saint Louis, MO, 63110, USA; 3 Division of Neuroradiology, Mallinckrodt Institute of Radiology, Washington University Medical Center, St. Louis, MO, 63110, USA; 4 Department of Radiation Oncology, Case Western University School of Medicine, 11100 Euclid Avenue, Cleveland, OH, 44106-6068, USA; 5 Department of Neurological Surgery, Washington University Medical School, 660 S. Euclid Avenue, Campus Box 8057, Saint Louis, MO, 63110, USA

## Abstract

**Introduction:**

Langerhans cell histiocytosis is a rare disease within the adult population, with very few cases reported as solitary hypophyseal lesions in adults. Of the reported cases, most have been treated successfully with surgery, radiotherapy, and/or chemotherapy. Radiotherapy has been thought to be curative at the relatively low dose of 20Gy. Here we report a case of recurrent hypophyseal Langerhans cell histiocytosis 9 months after radiotherapy with an interval period of symptomatic and radiographic response to therapy.

**Case presentation:**

A 50-year-old Caucasian woman who had headaches, memory difficulties, and diabetes insipidus was found to have a 2.5cm suprasellar mass. Langerhans cell histiocytosis was diagnosed following stereotactic brain biopsy. Further workup revealed no other lesions. Initial radiation treatment succeeded in shrinking the tumor and relieving clinical symptoms temporarily; however, growth and recurrence of clinical symptoms was noted at 9 months. Re-irradiation was well tolerated and the patient had no acute side effects.

**Conclusion:**

Isolated hypophyseal involvement by Langerhans cell histiocytosis in adults is a unique presentation of a rare disease. Although radiotherapy doses as low as 20Gy have been reported to offer control, this case demonstrates that higher doses may be warranted to ensure tumor control. With modern imaging and radiotherapy techniques higher doses should offer little increased more durable risk to surrounding critical structures.

## Introduction

Langerhans cell histiocytosis (LCH) is a rare disorder of antigen-presenting cells derived from the bone marrow that are termed ‘Langerhans cells’ in honor of their discovery by Paul Langerhans. The disease is believed to have first been reported in 1865 and has been known by many different names [1]. Early descriptions of disease in children with exophthalmos, lesions of skull bones, and diabetes insipidus (DI), led to the designation ‘Hand–Schüller–Christian disease’. Following the conceptualization of the reticuloendothelial system, patients with related disease that included hepatomegaly, splenomegaly, lymphadenopathy, and macrophage hyperplasia of various organs (among other symptoms) were known to have ‘Letterer–Siwe disease’. Reports of isolated ostotic lesions with prominent histiocytes have been referred to as ‘eosinophilic granulomas’ and ‘solitary granulomas of the bone.’ Isolated involvement of the hypothalamus was termed ‘Gagel’s granuloma’ [2]. In 1953, Lichtenstein unified these diseases as ‘Histiocytosis X’ with emphasis on patterns of clinical involvement. Subsequent electron microscopic characterization of Langerhans cells with the recognition of ‘Birbeck granules’ as their signature identifying feature, led to the eventual use of the current all-inclusive term LCH.

Although LCH is more common and best characterized in children, the disorder also presents in adults. It has an estimated incidence of 1 to 2 cases per million per year in adults with peak incidence at 33 years of age [3]. Clinical symptoms are related to the site of disease. Commonly affected organs include the lungs, skin, and bone; however, virtually any tissue may be involved, including the stomach, central nervous system, thyroid, and liver. Patterns of involvement are classified as single system, single system multifocal, and multisystem (disseminated). In general, better outcomes are observed in older patients and patients with single system disease; worse outcomes are observed in multisystem disease, in patients with organ dysfunction, in isolated pulmonary disease, and in younger patients [3]. Multisystem disease can be further classified as low risk or high risk (hematopoietic system, lungs, liver, and spleen) based on involvement of specific organs [4].

Endocrine abnormalities are relatively common in LCH multisystem disease. In one study, 81 of 188 (43.1%) adult patients with multisystem disease developed DI [3]. In the same study, no patient with single system disease developed DI. Hypothyroidism was also noted in 18 (9.6%) patients with multisystem disease and in no patients with single system disease. It is unclear whether these abnormalities in this series were attributable to pituitary and/or hypothalamic lesions or to compression of the pituitary stalk by nearby osseous lesions.

The ‘gold standard’ for histopathologic diagnosis is the presence of characteristic Birbeck granules on electron microscopy [5]. Other histologic features seen in LCH lesions include detection of CD1a, CD207, S100 protein, adenosine triphosphate (ATP)ase, or alpha-D-mannosidase on the cell surface [3]. In practice, the presence of CD1a and S100 on histiocytes is thought to be highly specific for LCH [6,7].

We present a case of isolated hypophyseal histiocytosis in a 50-year-old woman complicated by recurrence 9 months after initial radiation therapy. It rarely presents as an isolated lesion of the hypophysis. Of the previously reported cases we found in the literature, six were successfully treated with radiation therapy. This case was complicated by an early recurrence, suggesting that these tumors may require higher initial radiation doses, at least in adult presentations, to ensure good local control.

## Case presentation

A 50-year-old right-handed previously healthy Caucasian woman presented with a several month history of daily headaches, blurry vision, worsening daytime somnolence, and progressive memory difficulties. These symptoms had a significant impact on her activities of daily living and finally resulted in loss of employment. During this time she reported sleeping between 12 and 14 hours per day without improvement in energy level and had gained 18kg (40 pounds) in weight. She also reported a constant feeling of thirst, polydipsia (drinking 10 liters of fluid daily), polyuria, and nocturia. She had been amenorrheic for 5 years and reported no recent vaginal bleeding. She denied nausea, vomiting, heat or cold intolerance, and galactorrhea. Her past medical history was significant only for medication-controlled hypertension and she had no family history of endocrine abnormalities. Her physical examination was significant for bilateral peripheral visual field loss. The remainder of the examination, including a thorough neurological examination, was unremarkable. Laboratory studies demonstrated panhypopituitarism as follows (normal range in parentheses): thyroid-stimulating hormone 1.8uIU/mL (0.35–5.50), free thyroxine 0.52ng/dL (0.90–1.80), prolactin 42ng/mL (2.8–29.0), luteinizing hormone <1IU/L (postmenopausal range 15.9–54.0), follicle-stimulating hormone 2.1IU/L (23.0–116.3), adrenocorticotropic hormone 19pg/mL (0–49), insulin-like growth factor 38ng/mL (100–250), and cortisol at 4:00 p.m. <1.5mcg/dL (4.3–22.4).

**Figure 1:**
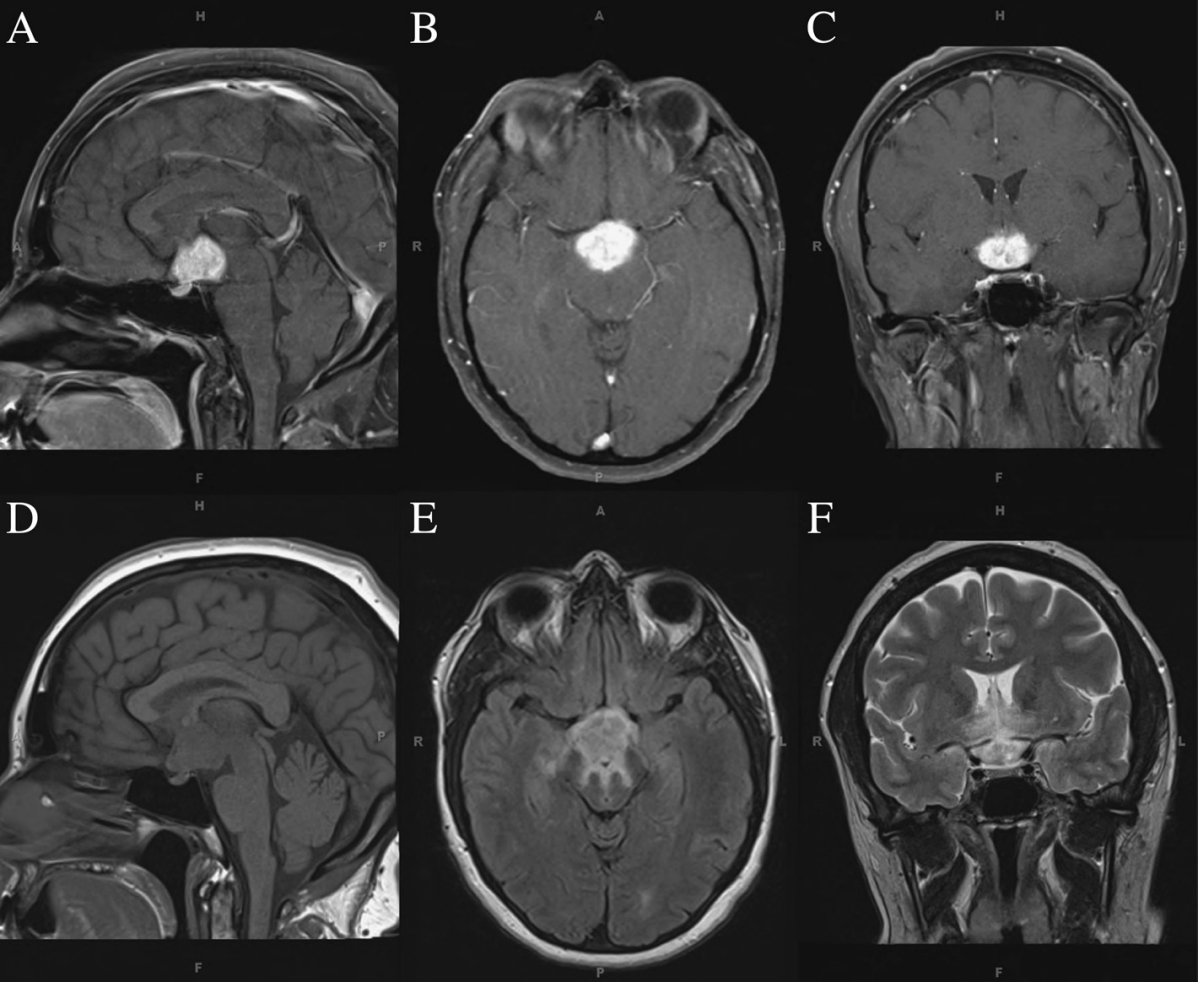
**Radiographic imaging at the time of initial diagnosis.** T1-weighted magnetic resonance image with (**A**, **B**, and **C**) and without (**D**) contrast demonstrate the large enhancing Langerhans cell histiocytosis lesion in the suprasellar region. Fluid attenuated inversion recovery (**E**) and T2-weighted imaging (**F**) demonstrate mild edema in the surrounding deep thalamic nuclei and brainstem.

A magnetic resonance image (MRI) of the patient’s brain with contrast showed a 25 × 22 × 20mm intensely enhancing suprasellar mass involving the hypothalamus and optic chiasm. The mass appeared to be separate from the pituitary on both sagittal and coronal imaging (Figure 1). Differential diagnosis included a metastatic lesion, as well as primary lesions such as central nervous system lymphoma, chordoma of the pituitary stalk, glioma, germinoma, sarcoidosis, and LCH. Computed tomography (CT) imaging of her chest, abdomen, and pelvis with contrast showed no evidence of neoplastic disease. Angiography revealed only mild displacement of the thalamic perforating arteries due to mass effect, and minimal tumor vascularity.

**Figure 2:**
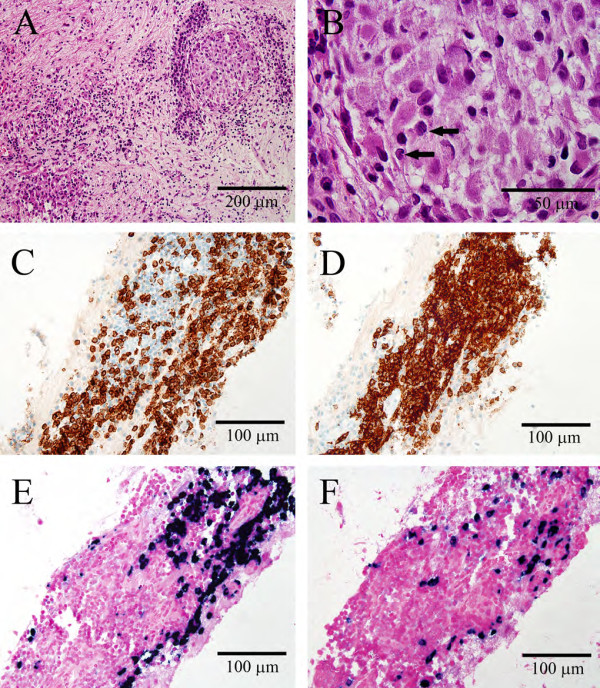
**Histological images of suprasellar stereotactic brain biopsy specimen.** Hematoxylin and eosin stained sections (**A** and **B**) show gliotic brain parenchyma with diffuse involvement by plasma cells, lymphocytes, and foci of histiocytes (**A**); high magnification of histiocytic aggregate shows cells with pale granular eosinophilic cytoplasm and grooved nuclei, indicated by arrows (**B**). Sections stained immunohistochemically for CD3 (**C**) and CD20 (**D**) show both T-cells and B-cells, respectively, within the lymphocytic infiltrates. *In situ* hybridization against kappa-light chain messenger ribonucleic acid (**E**) and lambda-light chain messenger ribonucleic acid (**F**) reveals a normal ratio of kappa-light chain to lambda-light chain expression. All histology images were captured using the following equipment: Olympus BX51 microscope, Olympus DP70 camera, and Olympus DP controller software. There was no image manipulation and scale bars were added in Photoshop, modeled over calibrated DP controller scale bars (not shown).

**Figure 3:**
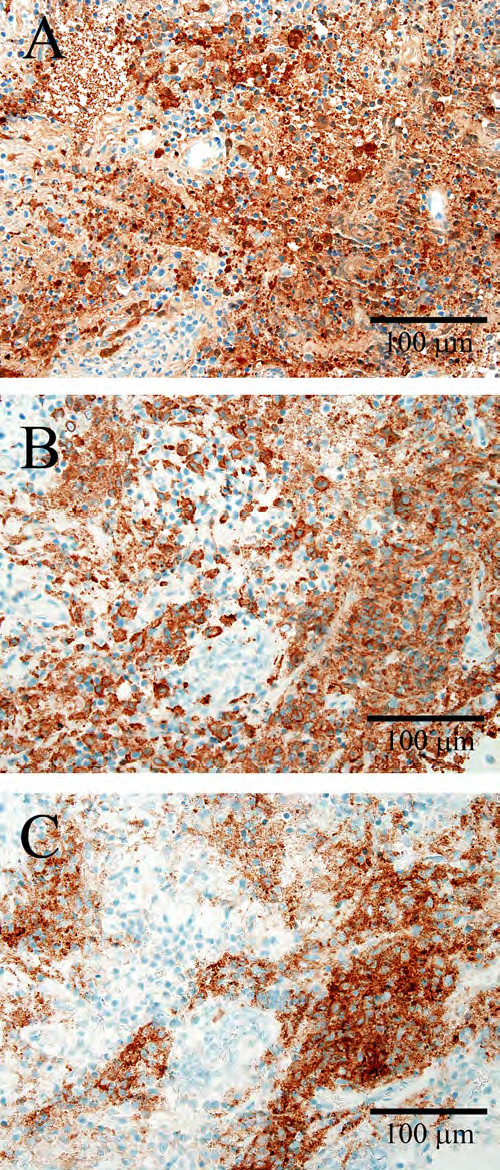
**Histological characterization of histiocytic cells.** Sections stained immunohistochemically show diffuse reactivity within histiocytes for S100 (**A**) and monocyte and macrophage marker CD163 (**B**), and a membranous staining pattern for CD1a (**C**).

A stereotactic needle biopsy of the mass was performed. A histological examination showed mildly gliotic brain parenchyma with a weakly angiocentric lymphoplasmacytic infiltrate that ranged from modest to intense. Also present were a few aggregates of histiocytes, one resembling a well-formed non-necrotizing granuloma (Figure 2A). Close inspection of these histiocytes revealed notched or grooved nuclei and abundant pale granular eosinophilic cytoplasm (Figure 2B). No atypical lymphocytes, no multinucleated giant cells, no infectious organisms or viral inclusions, and only rare eosinophils were identified. The histological differential diagnosis included: lymphoma, lymphocytic hypophysitis, and LCH. Immunohistochemical staining showed a mixed population of T-cells (Figure 2C) and B-cells (Figure 2D) with a normal 2:1 ratio of kappa- and lambda-light chain expression (Figure 2E and 2F), consistent with a reactive non-neoplastic infiltrate. Further characterization of the histiocytes showed strong reactivity for S100, CD163, and CD1a (Figure 3), supporting the diagnosis of LCH.

Prednisone and replacement hormone therapy, initiated immediately after her initial diagnosis, significantly improved the patient’s functional status; however, she remained fatigued and did not remember her initial hospital visits. Her case was presented at our multidisciplinary Tumor board. The consensus was that radiation therapy was a reasonable treatment approach for control of her tumor. She underwent CT simulation for radiation therapy planning. A 4-field 3D-conformal plan was generated with a prescription dose of 20Gy in 10 once-daily fractions. She completed radiation therapy with no acute complications.

At 3 months post-radiotherapy, she reported her energy level and cognition had significantly improved on endocrine therapy and at 5 months post-radiotherapy, an MRI demonstrated a marked decrease in the lesion size (8mm greatest dimension) with decreased edema in surrounding tissues as seen on fluid attenuated inversion recovery (FLAIR) imaging. At 9 months post-radiotherapy, she presented with a 2-week history of mild confusion, forgetfulness, increased fatigue, and an increase in weight. She has had routine follow-up visits with her endocrinologist with no recent changes in her endocrine replacement regimen. MRI at this time demonstrated an interval increase in lesion size from 8mm to 14mm and increased edema in the surrounding tissues.

Her case was presented at our multidisciplinary Neuro-Oncology Tumor board. Because she initially responded well to radiation therapy, a second course of radiation was recommended. She was re-simulated and treated with an additional 30Gy in 15 once-daily fractions. She completed radiotherapy with only mild fatigue. At the completion of treatment, she reported improvement in her vision and was able to reduce her steroid dose. Three months after completion of her second course of radiotherapy, an MRI demonstrated a decrease in size of the mass to 10mm with resolution of surrounding FLAIR signal, representing a favorable response to her second course of radiotherapy.

## Discussion

Overall, LCH is a rare disease in adults. As such, there is a paucity of data available to guide treatment of adult LCH. Depending on disease site and symptoms, therapy may include chemotherapy, immune modulation, radiation therapy or observation. An ongoing trial evaluating chemotherapy is being conducted by the Histiocyte Society (trial LCH-A1), but results have yet to be published. In 2003 the Histiocyte Society published a retrospective analysis of 274 adults in 13 countries with LCH [3]. Chemotherapy, most often vinblastine with or without steroids, was the initial treatment in 138 patients. Immunotherapy with prednisone or cyclosporine (n = 21) or observation (n = 110) were also initial therapeutic options. There was no reported analysis of outcomes based on chosen treatment modality. Another group reported results of seven patients treated with MACOP-B (methotrexate, arabinofuranosyl cytidine [cytarabine], cyclophosphamide, Oncovin® [vincristine], prednisone, and bleomycin), a chemotherapy regimen used for aggressive lymphoma [8]. All patients showed a response; complete response (CR) was observed in five patients. CR was maintained for 2 patients at a median follow-up of 6.5 years. Relapse was observed in the other patients at 5, 8, and 62 months. In children, a study of 170 single system (e.g. low-risk) LCH patients showed that 81% of patients were disease free after initial therapy [9]. A second pediatric trial of 143 multisystem (e.g. high-risk) LCH patients were randomized to vinblastine or etoposide for 24 weeks with a single initial dose of corticosteroids [10]. Response was equivalent (76% and 83% 3-year-survival respectively). Demographic parameters known to be correlated with improved survival included: age >2 years; fewer involved organs; and response at 6 weeks.

To date, only six cases of isolated pituitary lesions in adults treated with radiotherapy have been described in the literature [11-19]. The five most recent cases were treated with 20Gy in 10 fractions (three patients) [15,16], 21Gy in 15 fractions (one patient) [11], 21Gy with the addition of chemotherapy (one patient) [19], or radiosurgery (one patient) [17]. Fractionated treatment resulted in stabilization or shrinkage of disease with reported follow-up ranging from 1 to 8 years; no failures have been reported to date. The patient treated with radiosurgery experienced recurrence approximately 1 year after treatment. His recurrence was successfully controlled with vinblastine, cyclophosphamide and prednisone. For the patient in our report, a dose of 30Gy was selected. We wished to take the lesion to a cumulative radiation dose below the threshold for radiation-induced toxicity to the optic nerves and chiasm, but significantly higher than that received in her prior course of radiotherapy. The increased fraction number, higher dose and smaller initial lesion size would suggest a greater chance of control from a radiobiological standpoint.

There are reports suggesting the use of chemotherapy as a first-line treatment for these patients [18,19]. This treatment strategy was discussed both on initial presentation and at the time of recurrence in this case. At the time of initial diagnosis, it was felt that local therapy could offer a durable response with minimal risk of side effects from radiotherapy while reserving chemotherapy for treatment of systemic disease, if it were to arise. At the time of recurrence, the use of chemotherapy was revisited. Based on the initial good response of the tumor to fractionated therapy and the lack of any systemic disease, a second course of radiation was prescribed. As chemotherapeutic agents commonly used to treat LCH (vinca alkaloids, antimetabolites, and antifolates) are often associated with significant side effects, it was felt chemotherapy would be reserved for salvage therapy when either surrounding normal tissues would not tolerate re-irradiation or if systemic disease were to arise. Re-irradiation of any site is more technically challenging and may carry increased risk of adverse side effects compared to initial therapy. The fact that the patient was re-treated at the same institution using modern CT simulation techniques mitigated some technical difficulties and allowed us to calculate composite dose distributions, thus enabling us to ensure that critical structures were spared. The QUANTEC (Quantitative Analyses of Normal Tissue Effects in the Clinic) data analysis on radiation-induced toxicities was used to evaluate risk to critical structures based on the composite plan. All parameters were well within the minimal risk parameters. Specifically, radiation doses less than 60Gy in 2Gy fractions to the optic nerves and chiasm have a very low rate of radiation-induced optic neuritis [20]. The optic chiasm was adjacent to the radiation field and received a total (composite) mean dose of 51.43Gy (total max dose 52.10Gy). The optic nerves received slightly lower mean doses than the chiasm in the composite plan, 13.53 and 9.36, and lower maximum point doses, 48.94Gy and 43.76Gy, for the left and right respectively. The brain and brainstem were also well within the allowed limits for minimal risk with median composite doses of 12.34 and 32.42, and maximum composite doses of 52.25Gy and 52.09Gy, respectively [21,22].

## Conclusion

This case demonstrates a rare presentation of a rare disease. Although a handful of cases involving LCH limited to the hypothalamic region have been reported, this one represents only the second example of recurrence after radiation therapy. Given the location of this disease, a higher initial radiotherapy dose may be justified because this will probably reduce the chance of failure in a region of the brain surrounded by multiple critical structures. Higher radiotherapy doses are easily achievable in this region using modern radiotherapy techniques that maintain minimal risk of radiation-related toxicities.

## Consent

Written informed consent was obtained from the patient for publication of this case report and accompanying images. A copy of the written consent is available for review by the Editor-in-Chief of this journal.

## Competing interests

The authors declare that they have no competing interests*.*

## Authors’ contributions

The patient was under the care of MC and JRS. Pathology was reviewed by RP and KH. Imaging was reviewed by JJS. DF, RF, DM and JJ contacted the patient for consent and drafted the manuscript. RF and DF contributed equally to this manuscript. All authors reviewed and approved the manuscript prior to publication.
